# Ginseng Is Useful to Enhance Cardiac Contractility in Animals

**DOI:** 10.1155/2014/723084

**Published:** 2014-02-04

**Authors:** Jia-Wei Lin, Yih-Giun Cherng, Li-Jen Chen, Ho-Shan Niu, Chen Kuei Chang, Chiang-Shan Niu

**Affiliations:** ^1^Department of Neurosurgery, Taipei Medical University-Shuang Ho Hospital and College of Medicine, Taipei Medical University, Taipei 10361, Taiwan; ^2^Department of Anesthesiology, Taipei Medical University-Shuang Ho Hospital and College of Medicine, Taipei Medical University, Taipei 10361, Taiwan; ^3^Institute of Basic Medical Sciences, College of Medicine, National Cheng Kung University, Tainan 70101, Taiwan; ^4^Department of Nursing, Tzu Chi College of Technology, Hualien City 97005, Taiwan

## Abstract

Ginseng has been shown to be effective on cardiac dysfunction. Recent evidence has highlighted the mediation of peroxisome proliferator-activated receptors (PPARs) in cardiac function. Thus, we are interested to investigate the role of PPAR**δ** in ginseng-induced modification of cardiac contractility. The isolated hearts in Langendorff apparatus and hemodynamic analysis in catheterized rats were applied to measure the actions of ginseng *ex vivo* and *in vivo*. In normal rats, ginseng enhanced cardiac contractility and hemodynamic *dP*/*dt*_max_ significantly. Both actions were diminished by GSK0660 at a dose enough to block PPAR**δ**. However, ginseng failed to modify heart rate at the same dose, although it did produce a mild increase in blood pressure. Data of intracellular calcium level and Western blotting analysis showed that both the PPAR**δ** expression and troponin I phosphorylation were raised by ginseng in neonatal rat cardiomyocyte. Thus, we suggest that ginseng could enhance cardiac contractility through increased PPAR**δ** expression in cardiac cells.

## 1. Introduction

Ginseng varieties have been garnering increasing interest recently for their effects on the cardiovascular system [1]. It has been demonstrated that ginseng could prevent cardiac hypertrophy and heart failure through a mechanism likely involving the prevention of calcineurin activation [2] and the latter representing a key factor for myocardial hypertrophy and remodeling [3, 4].

Peroxisome proliferator-activated receptors (PPARs) are ligand-activated transcriptional factors that regulate the expression of genes involved in lipid metabolism and inflammation [5]. Three subtypes of PPARs, PPAR*α*, PPAR*γ*, and PPAR*δ*, have been shown to modulate the expressions of various genes to exert bioactivity [5]. PPAR*α* is relatively abundant in tissues with a high oxidative capacity, such as the liver and heart, whereas PPAR*γ* is confined to a limited number of tissues, primarily adipose tissue [5, 6]. The ubiquitously expressed PPAR*δ* enhances lipid catabolism in adipose tissue and muscles [5], and PPAR*δ*-dependent maintenance of inotropic function is crucial for cardiomyocytes [7–9]. Deletion of cardiac PPAR*δ* is accompanied by decreased contraction, increased left ventricular end-diastolic pressure, and lowered cardiac output and leads to decreased contraction and increased incidence of cardiac failure [7].

It has been identified that cardiac agent, such as digoxin and dobutamine, can restore the cardiac contractility in diabetic rats [10–12]. Also, cardiac agent improved cardiac contraction in STZ-diabetic rats which is associated with a marked increase in cardiac PPAR*δ* expression [13].

Thus, we are interested to screen the effect of ginseng on cardiac contractility and investigate the mediation of PPAR*δ* in this action of ginseng. Using the isolated hearts and animals in addition to cultured cardiac cells, the main aim of the present study is to clarify if ginseng can enhance cardiac contractility through increased PPAR*δ* expression or not.

## 2. Materials and Methods

### 2.1. Materials

GSK0660 (a specific PPAR*δ* antagonist) was purchased from Santa Cruz Biotechnology, Inc. (Santa Cruz, CA, USA). The fluorescent probe, fura-2, was the product of Molecular Probes (Eugene, OR, USA). Antibodies specific to PPAR*δ*, cardiac troponin I (TnI) or phospho-troponin I (p-TnI) (Ser 23/24), were obtained from Cell Signaling Technology (Beverly, MA, USA).

### 2.2. Preparation of Fermented *Panax ginseng* (*P. ginseng*)

*Panax ginseng* extract (GINST) used in the present study was provided by Bing-Han Pharmaceutics (Hsin-Yin, Tainan Shang, Taiwan). Briefly, dried *Panax ginseng* (1 kg) was extracted with 5 L of 50% aqueous ethanol at 85°C and concentrated in vacuous to obtain dark brown syrup. The dark brown syrup then was mixed with starch to generate the ginseng powder. The powder containing 95% *P. ginseng* root and 5% starch was dissolved in saline solution for oral administration at the desired doses.

### 2.3. Animals

Male Wistar rats, weighing from 200 to 250 g, were obtained from the Animal Center of National Cheng Kung University Medical College. All experiments were performed under anesthesia with 2% isoflurane and all efforts were made to minimize suffering. The animal experiments were approved and conducted in accordance with local institutional guidelines for the care and use of laboratory animals in Chi-Mei Medical Center (no. 100052307) and performed according to the Guide for the Care and Use of Laboratory Animals as well as the guidelines of the Animal Welfare Act.

### 2.4. Drug Administration

Animals were randomly assigned into three groups: (I) the control group (*n* = 8) treated with the vehicle, saline (0.9% sodium chloride, orally); (II) the ginseng (Gin) group (orally. *n* = 8) treated by oral administration with ginseng powder at 400 mg/kg for 7 days as described previously [14], and (III) the ginseng + GSK0660 (Gin + GSK) group (*n* = 8) treated with ginseng powder at effective dose (400 mg/kg, orally) according to previous report [14] and GSK0660 at effective dose (3 mg/kg, i.v.) [15] for 7 days. At the end of experiment, hearts of each group were dissected out for Western blotting analysis and Real-time reverse transcription-polymerase chain reaction.

### 2.5. Langendorff Apparatus for Isolated Heart Determination

The experiment was performed according to a previous description [16]. The rats were sacrificed under anesthesia with 3% isoflurane and their hearts were excised rapidly and rinsed by immersion in ice-cold Krebs-Henseleit buffer (KHB) (mM: NaCl 118.5, KCl 4.7, MgSO_4_ 1.2, CaCl_2_ 1.8, NaHCO_3_ 25.0, and glucose 11.0 at pH 7.35). The hearts were mounted in the Langendorff apparatus and continuously perfused with warm (37°C) and oxygenated (95% O_2_, 5% CO_2_) KHB at a constant pressure of 70 mmHg. The organ chamber temperature was maintained at 37°C during the experiment. A water-filled latex balloon was inserted through an incision in the left atrium into the left ventricle via the mitral valve and adjusted to a left ventricular end-diastolic pressure (LVEDP) of 5–7 mmHg during initial equilibrium. The distal end of the catheter was connected to an iWorx 214 TM data acquisition system (Ladscrib 2.0 software, iWorx Systems, Inc., Dover, NH, USA) via a pressure transducer for continuous recording. Left ventricular systolic function was assessed by recording the left ventricular developed pressure (LVDP), which was defined as the difference between left ventricular end-systolic pressure (LVESP) and LVEDP.

### 2.6. Catheterization for Hemodynamic *dP*/*dt* Measurement

This part of experiments was performed in rats under anesthesia with 2% isoflurane to the minimize suffering of animals. Temporary pacing leads were used for the short-term study and were placed in the right atrium and RV apex. A venogram imaged in 2 different angulations (left anterior oblique 30° and anteroposterior) was obtained to determine the anatomy of the coronary sinus venous system. An LV pacing electrode (IX-214; iWorx Systems, Inc., Dover, NH, USA) was placed either in the free wall region via the lateral or posterior vein or in the anterior region via the great cardiac vein. After femoral artery and venous puncture using the Seldinger technique [17], pressure transducer catheters were inserted into the heart to provide the RV, aortic, mean blood, and LV pressures. Pressure catheters and pacing leads were connected to an external pacing computer (iWorx Systems, Inc., Dover, NH, USA) to monitor the heart rate and to acquire hemodynamic signals. Body temperature of the rats was also maintained at 37.5°C throughout whole procedure.

### 2.7. Cell Cultures

Primary cultures of neonatal rat cardiomyocytes were prepared by the modification of a previously described method [18]. Briefly, under anesthesia with 3% isoflurane, the hearts of 1- to 2-day-old Wistar rats were excised, cut into 1–2 mm pieces, and predigested with trypsin to remove red blood cells. The heart tissue was then digested with 0.25% trypsin and 0.05% collagenase. The dissociated cells were placed in uncoated 100 mm dishes and incubated at 37°C in a 5% CO_2_ incubator for at least 1 h to remove the nonmyocytic cells. This procedure caused fibroblasts to predominantly attach to the dishes while most of the cardiomyocytes remained in suspension. The cardiomyocyte-enriched population was then collected and counted. The cells were cultured in Dulbecco/Vogt modified Eagle's minimal essential medium (DMEM) with 1 mmol/L pyruvate, 10% fetal bovine serum, 100 units/mL penicillin, and 100 units/mL streptomycin. Over 95% of the collected cells were characterized as cardiomyocytes on the basis of the sarcomeric myosin content. On the second day, the medium was replaced. After 3 to 4 days in culture, the cells were used in the experiments. Stock solutions of ginseng and GSK0660 were prepared with DMSO (0.1%). The cells were treated with 100 *μ*g/mL ginseng for 24 h [14], washed twice with PBS, and removed by trypsinization. The cells were then collected and subjected to a protein expression assay. Additional treatments with GSK0660 (10^−6 ^M) [19] were performed for 30 minutes before the ginseng treatment.

### 2.8. Measurement of Intracellular Calcium Concentration

The changes in intracellular calcium were detected using the fluorescent probe fura-2-AM [20]. The neonatal rat cardiomyocytes were placed in buffered physiological saline solution (PSS) containing 140 mM NaCl, 5.9 mM KCl, 1.2 mM CaCl_2_, 1.4 mM MgCl_2_, 11.5 mM glucose, 1.8 mM Na_2_HPO_4_, and 10 mM Hepes-Tris, to which was added 5 *μ*M fura-2-AM, and the solution was incubated for 1 h in humidified 5% CO_2_ and 95% air at 37°C. The cells were washed and incubated for an additional 30 minutes in PSS. The neonatal rat cardiomyocytes were inserted into a thermostatic (37°C) cuvette containing 2 mL of calcium-free PSS. After recording the baseline value, ginseng (100 *μ*g/mL) was added into the cuvette with/without GSK0660 (10^−6 ^M) to detect the free intracellular calcium. The fluorescence was continuously recorded using a fluorescence spectrofluorometer (Hitachi F-2000, Tokyo, Japan). Values of [Ca^2+^]i were calculated from the ratio *R* = F340/F380 by the formula [Ca^2+^]i = *KdB* (*R* − *R*_min⁡_)/(*R*_max⁡_ − *R*), where *Kd* was 225 nM, F was fluorescence, and *B* was the ratio of the fluorescence of the free dye to that of the Ca^2+^-bound dye measured at 380 nm. *R*_max⁡_ and *R*_min⁡_ were determined in separate experiments by using ginseng to equilibrate [Ca^2+^]j with ambient [Ca^2+^] (*R*_max⁡_) and the addition of 0.1 mM MnCl_2_ and 1 mmol/L EGTA (*R*_min⁡_). Background autofluorescence was measured in unloaded cells and subtracted from all measurements.

### 2.9. Western Blotting Analysis

Protein was extracted from tissue homogenates and cell lysates using ice-cold radio-immunoprecipitation assay (RIPA) buffer supplemented with phosphatase and protease inhibitors (50 mmol/L sodium vanadate, 0.5 mM phenylmethylsulphonyl fluoride, 2 mg/mL aprotinin, and 0.5 mg/mL leupeptin). Protein concentrations were determined with a Bio-Rad protein assay (Bio-Rad Laboratories, Inc., Hercules, CA, USA). Total proteins (30 *μ*g) were separated by SDS/polyacrylamide gel electrophoresis (10% acrylamide gel) using a Bio-Rad Mini-Protein II system. The protein was transferred to expanded polyvinylidene difluoride membranes (Pierce, Rockford, IL, USA) with a Bio-Rad Trans-Blot system. After transfer, the membranes were washed with PBS and blocked for 1 h at room temperature with 5% (w/v) skimmed milk powder in PBS. The manufacturer's instructions were followed for the primary antibody reactions. Blots were incubated overnight at 4°C with an immunoglobulin-G polyclonal rabbit anti-mouse antibody (Affinity BioReagents, Inc., Golden, CO, USA) (1 : 500) in 5% (w/v) skimmed milk powder dissolved in PBS/Tween 20 (0.5% by volume) to bind the target protein such as PPAR*δ*. The blots were incubated with goat polyclonal antibody (1 : 1000) to bind the actin, which served as the internal control. After the removal of the primary antibody, the blots were extensively washed with PBS/Tween 20 and then incubated for 2 h at room temperature with the appropriate peroxidase-conjugated secondary antibody diluted in 5% (w/v) skimmed milk powder and dissolved in PBS/Tween 20. The blots were developed by autoradiography using an ECL-Western blotting system (Amersham International, Buckinghamshire, UK). The immunoblots of PPAR*δ* (50 kDa), cardiac troponin I (28 kDa), and phospho-troponin I (28 kDa) were then quantified using a laser densitometer.

### 2.10. Statistical Analysis

Results were expressed as mean ± SE of each group. Statistical analysis was carried out using ANOVA analysis and Newman-Keuls post hoc analysis. Statistical significance was considered at *P* < 0.05.

## 3. Results

### 3.1. Effect of GW0742 on PPAR*δ* Expression and TnI Phosphorylation in the Heart of Rats

**Figure 1:**
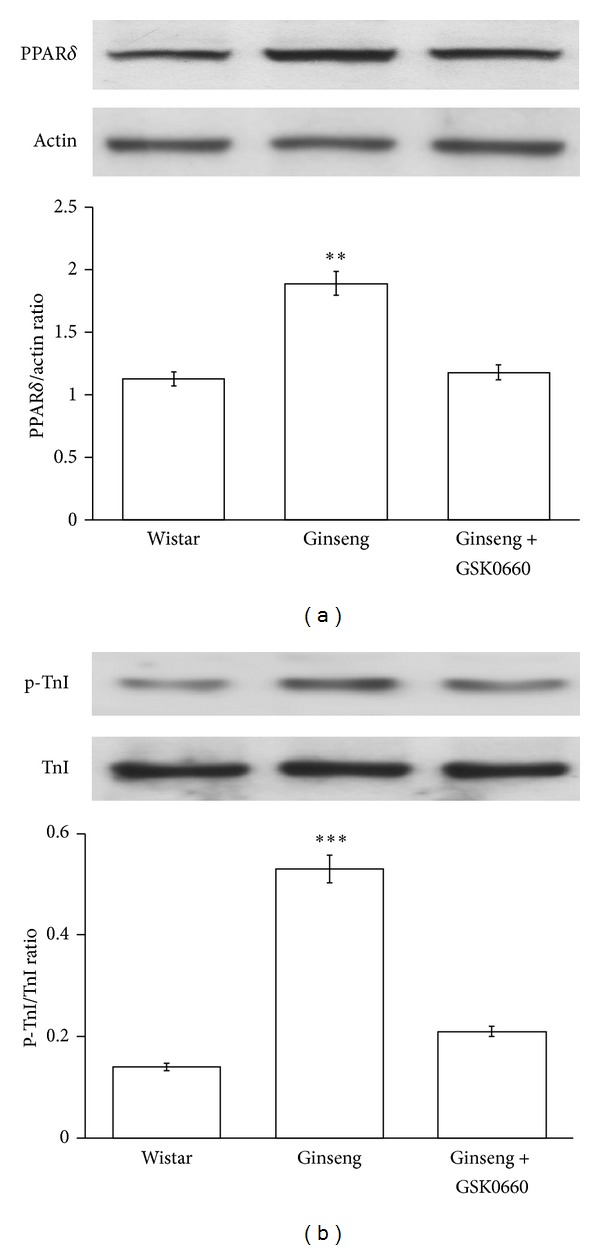
Effects of ginseng on PPAR*δ* expression and TnI phosphorylation in heart of rats. Changes of PPAR*δ* expression (a) and TnI phosphorylation (b) in the hearts of rats treated with ginseng. Rats were treated with ginseng (400 mg/kg) for 7 days and hearts were then used to measure the protein level of PPAR*δ* expression and TnI phosphorylation using Western blotting analysis. All values are presented as mean ± SEM (*n* = 8). **P < 0.01 and ***P < 0.001 compared with normal rats.

The rats treated with ginseng were used to identify changes in PPAR*δ* expression and TnI phosphorylation. The level of PPAR*δ* expression (Figure 1(a)) and TnI phosphorylation (Figure 1(b)) was markedly raised by ginseng at an effective concentration (400 mg/kg). In addition, this change was reversed by GSK0660 (3 mg/kg, i.v.) at a concentration that did not modify the level of PPAR*δ* expression and TnI phosphorylation (Figure 1).

### 3.2. Effect of Ginseng on Cardiac Performance in the Isolated Rat Heart

**Figure 2:**
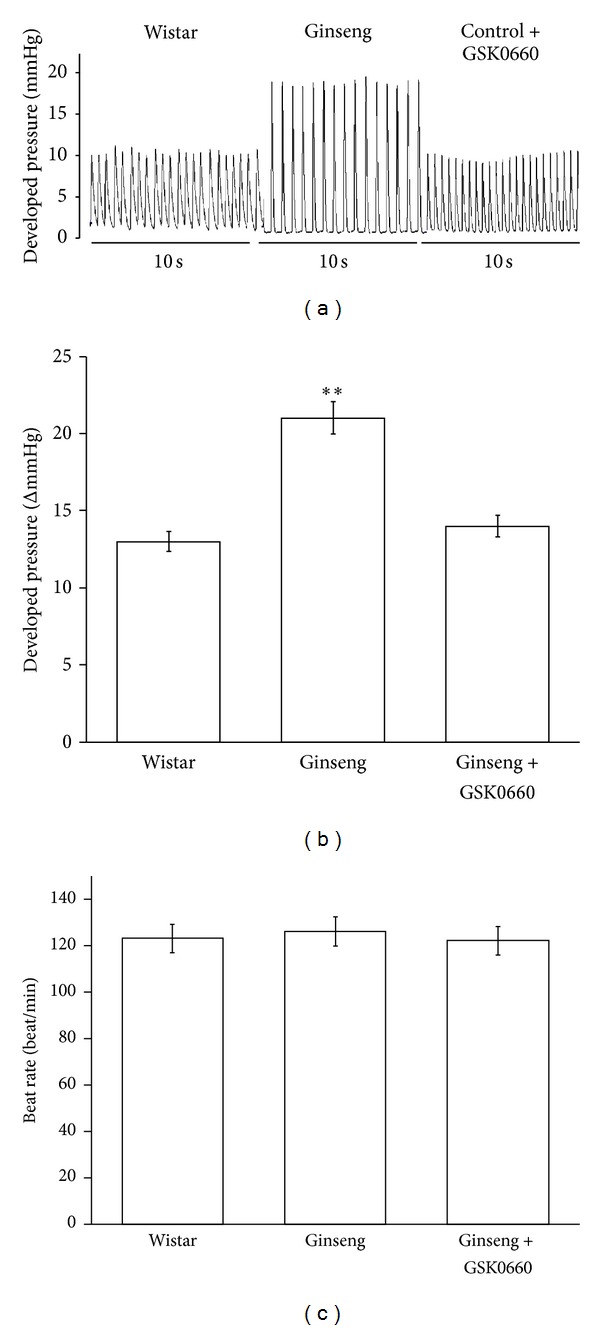
Effects of ginseng on cardiac performance in hearts isolated from rats. The representative picture shows the change in cardiac performance caused by ginseng in isolated hearts (a). Heart rate and cardiac contractility were recorded in isolated rat heart treated with ginseng or cotreated with ginseng + GSK0660. The changes in developed pressure (b) and beat rate (c) were recorded continuously throughout the whole experiment. All values are presented as mean ± SEM (*n* = 8). **P < 0.01 as compared to normal rats.

Ginseng at a sufficient dose to activate PPAR*δ* was used to treat the hearts isolated from rats. As shown in Figure 2, ginseng (100 *μ*g/mL) increased cardiac contractility without changes in heart rate. Moreover, the cardiac tonic action of ginseng was diminished by GSK0660 (10^−6 ^M) (Figure 2).

### 3.3. Effect of Ginseng on Cardiac Performance in the Anesthetized Rats

**Figure 3:**
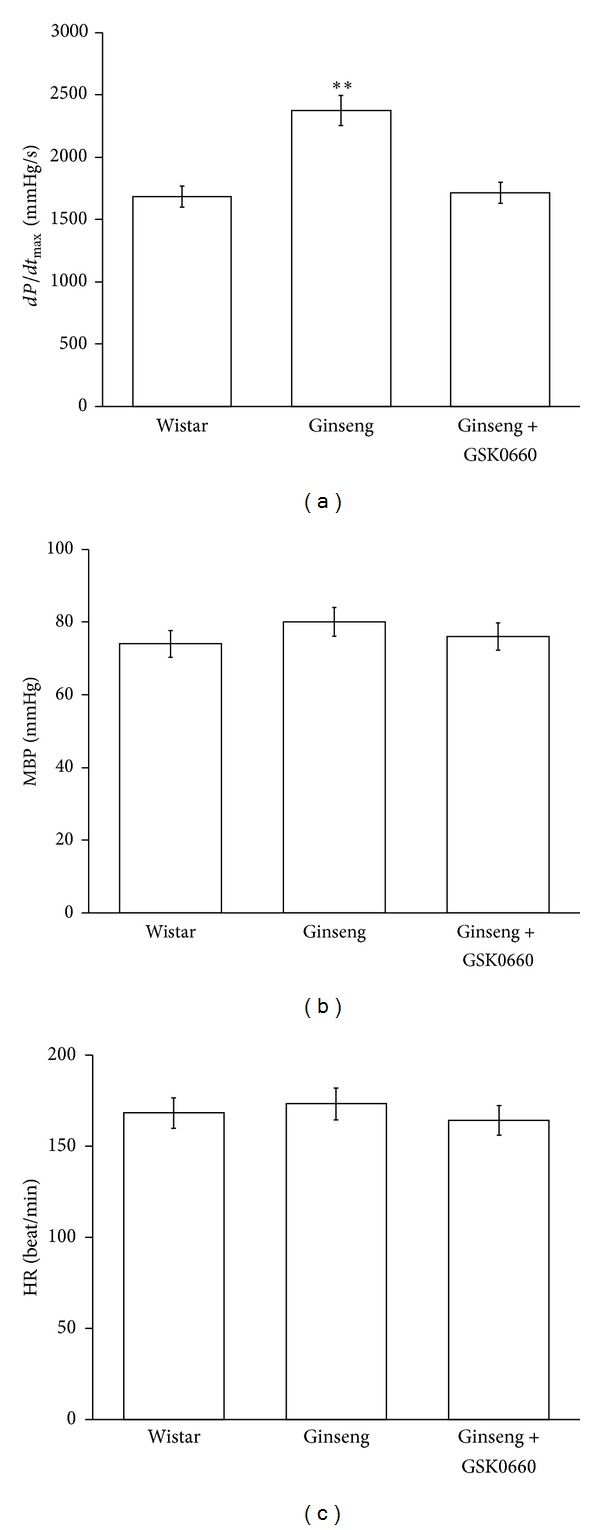
Effects of ginseng on cardiac performance in anesthetized rats. The effects of coadministration of ginseng and/or GSK0660 were investigated in the anesthetized rats. The changes in hemodynamic *dP*/*dt* (a), mean blood pressure (MBP) (b), and heart rate (HR) (c) were recorded continuously throughout the whole experiment. All values are presented as mean ± SEM (*n* = 8). **P < 0.01 as compared to normal rats.

The *dP*/*dt*_max⁡_ was significantly increased by ginseng after the treatment (400 mg/kg/day, orally for 7 days) as described previously [14] in the anesthetized rats, compared with the vehicle-treated control. However, this effect disappeared in the rats receiving coadministration of GSK0660 at effective dose (3 mg/kg, i.v.) [15] (Figure 3(a)). Treatment of ginseng only did not modify the heart rate but produced a slight increase in blood pressure that was also blocked by GSK0660 (Figures 3(b) and 3(c)).

### 3.4. Effect of Ginseng on Intracellular Calcium in Neonatal Rat Cardiomyocytes

**Figure 4:**
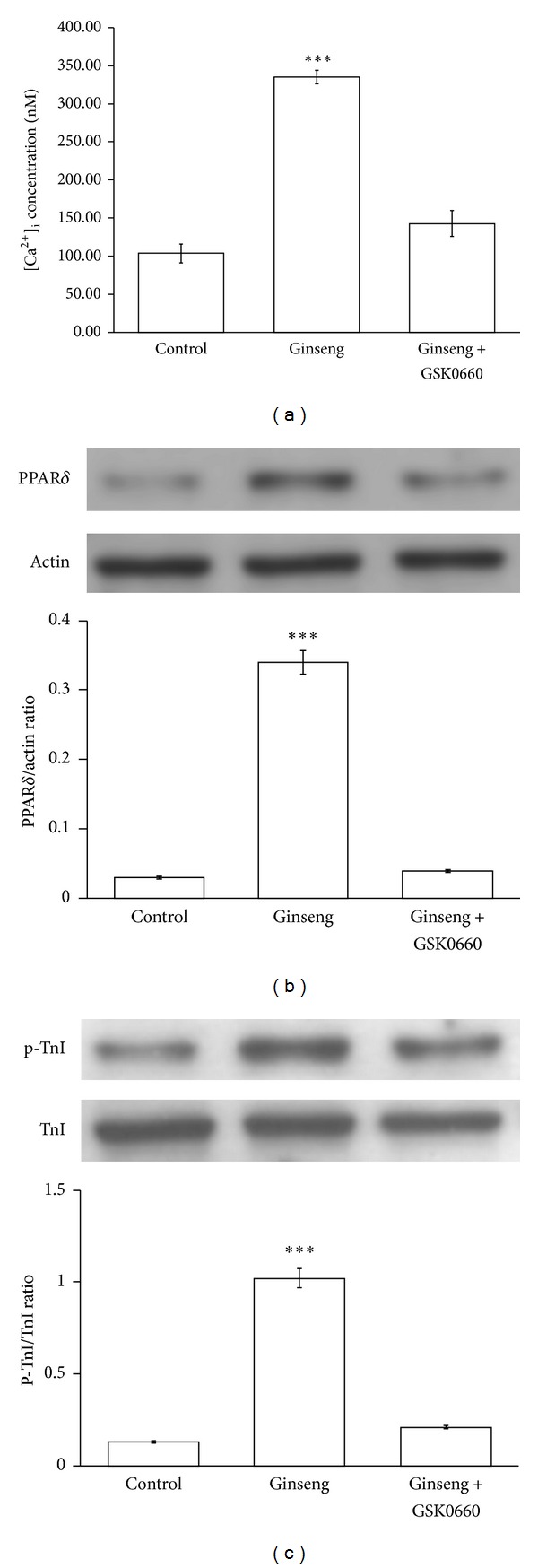
Effects of ginseng on intracellular calcium and TnI phosphorylation in neonatal rat cardiomyocytes. Changes in intracellular calcium were detected with fura-2 by using a fluorescence spectrofluorometer. The neonatal rat cardiomyocytes were placed in buffered physiological saline solution with 5 *μ*M of fura-2-AM and incubated for 1 h. After recording the baseline value, ginseng was added into the cuvette with/without GSK0660 to detect the free intracellular calcium (a). Effects of ginseng on PPAR*δ* expression (b) and TnI phosphorylation (c) in the neonatal rat cardiomyocytes were indicated. Cells treated with ginseng for 24 hours were harvested to measure the protein level of PPAR*δ* expression and TnI phosphorylation using Western blotting analysis. All values are presented as mean ± SEM (*n* = 8). ***P < 0.001 compared with the control group.

The fluorescent probe, fura2-AM, was used to detect changes in intracellular calcium level in the neonatal rat cardiomyocytes, and ginseng at an effective concentration (100 *μ*g/mL) was found to increase the intracellular calcium level. This effect disappeared in the cardiomyocytes that were coincubated with ginseng and GSK0660 (10^−6 ^M) (Figure 4(a)); however, incubation with GSK0660 alone did not affect the intracellular calcium level in the neonatal rat cardiomyocytes (Figure 4(a)).

### 3.5. Effects of Ginseng on PPAR*δ* Expression and TnI Phosphorylation in Neonatal Rat Cardiomyocytes

The neonatal rat cardiomyocytes were applied to treat with ginseng for identification of changes in PPAR*δ* expression and TnI phosphorylation. The levels of PPAR*δ* expression and TnI phosphorylation were markedly raised by ginseng at an effective concentration (100 *μ*g/mL). Also, this effect was reversed by GSK0660 (10^−6 ^M) at a concentration that did not modify the level of PPAR*δ* expression and TnI phosphorylation (Figure 4(b)).

## 4. Discussion

In the present study, we found that ginseng increased cardiac contractility but not heart rate in rats at the dose of 400 mg/kg, orally. This dose is equal to human oral dose about 3871 mg/kg by using the US FDA HED (human equivalent dose) equation for calculation [21–23]. Increases in PPAR*δ* expression and TnI phosphorylation were also observed in the heart of ginseng-treated rats. In hearts isolated from rats, ginseng enhanced cardiac contractility and this action was diminished by GSK0660 at a concentration sufficient to block PPAR*δ*. In the anesthetized rats, cardiac contraction (*dP*/*dt*_max⁡_) was also significantly increased by ginseng and this change was blocked by GSK0660. However, heart rate was not modified by ginseng at same dose. In the neonatal rat cardiomyocytes, ginseng increased cellular calcium levels, PPAR*δ* expression, and TnI phosphorylation. Thus, to the best of our knowledge, this is the first study to show that ginseng could increase cardiac contractility through activation of PPAR*δ*.

*In vivo* and *in vitro* investigations have revealed a number of significant actions of ginsenosides and ginseng extracts in cardioprotection, such as reducing myocardial ischemia-reperfusion induced damage via NO pathway in rats and mice [24], slowing down deterioration of cardiac contractions, preventing the development of arrhythmias [25], and relaxing the muscles of the aorta [26]. Also, it has been documented that ginseng increases cardiac lipid metabolism by enhancement of PPAR*δ* expression in the hearing [27]. In this study, we found that ginseng could increase PPAR*δ* expression and TnI phosphorylation. Also, this action of ginseng was abolished by specific PPAR*δ* antagonist. Mediation of PPAR*δ* in this action of ginseng can thus be considered.

It has been established that PPAR*δ* plays an important role in the regulation of cardiac performance [17–19]. In this study, we demonstrated that ginseng increases cardiac contractility without affecting heart rate. Also, this cardiac action of ginseng is reversed by blockade of PPAR*δ* using antagonist. Furthermore, activation of PPAR*δ* using ginseng enhanced cardiac contractility in the isolated hearts and the hemodynamic *dP*/*dt*_max⁡_ in the rats. Both actions of ginseng were inhibited by GSK0660 at a concentration sufficient to block PPAR*δ* [27, 28]. The enhancement of cardiac contractility by ginseng through an activation of PPAR*δ* is then characterized.

A change in heart rate is the most serious side effect of cardiac agents [29, 30]. In the present study, we showed that ginseng could enhance cardiac contractility without altering heart rate in isolated heart. In addition, ginseng generated cardiac tonic action in animals without impacting the heart rate. Thus, ginseng can be used as cardiac agent at some doses without side effect of arrhythmia. Our previous studies have showed that activation of PPAR*δ* by cardiac agent may improve diabetic cardiomyopathy in type-1 diabetic rats [28, 31]. Thus, ginseng seems helpful in the treatment and/or prevention of diabetic cardiomyopathy. However, a slight elevation of mean blood pressure was observed in the rats received ginseng. Thus, applying ginseng in patients with hypertension should be done carefully.

Troponin I (TnI) is known as an inhibitory unit of the troponin complex associated with thin filaments, and it inhibits actin-myosin interactions at the diastolic level of intracellular Ca^2+^ [32, 33]. Modulation of myofilament properties by alterations in TnI phosphorylation has been found to have a profound effect on cardiac contractility [34]. Phosphorylation of TnI has been shown to increase the cross-bridge cycling rate, leading to an increase in power output [33, 34]. Ca^2+^ is mainly involved in muscle contraction [32–35]. Contraction of cardiac muscles relies upon interactions between ATP and Ca^2+^, both of which must be present in adequate amounts [36]. We observed that ginseng has the ability to increase the amount of intracellular calcium in cardiomyocytes, and this seemed to be related to the higher contractility of heart.

It has been shown that TnI phosphorylation most likely acts through an enhanced off rate during Ca^2+^ exchange with contractile protein, leading to an increase in cardiac output [36–40]. Consistent with this, we found that TnI phosphorylation was elevated in the neonatal rat cardiomyocytes exposed to ginseng. Thus, direct activation of PPAR*δ* by ginseng may result in a higher level of TnI phosphorylation. Both changes caused by ginseng in the cardiomyocytes may explain the increase in cardiac contractility.

The multiple cell signals are involved in cardiac contractility. It is not easy to speculate the potential mechanism(s) for this action of ginseng in the increase of cardiac contractility. It has been documented that ginsenoside, one of the active principles in ginseng, can improve systolic/diastolic function and enhance cardiac *dP*/*dt* through the opening of mitochondrial adenosine triphosphate-sensitive potassium channels [41], reduction in oxidative stress via inhibition of glutathione [42], and augmentation of cellular calcium influx [43]. The increase of cellular calcium influx is consistent with to our previous reports regarding PPAR*δ* related cardiac tonic actions [10, 11, 44].

Interestingly, ginseng enhanced cardiac contractility without altering heart rate in isolated heart. Also, ginseng generated cardiac tonic action in animals without impacting the heart rate. It has been demonstrated that PPAR*δ* agonist is not effective in cardiac conduction [45]. The possible explanation for this seems related to the absence of PPAR*δ* in cardiac conduction system. However, the real mechanism(s) for the lack of effect on heart rate caused by ginseng require(s) more investigations in the future.

The activation of PPAR*δ* by ginseng can enhance the cardiac contractility without altering heart rate. Thus, we suggest that ginseng is suitable for the treatment of heart failure. Using this agent, arrhythmia can be ignored in patients for treatment of heart failure.

## 5. Conclusion

According to these findings, we suggest that the activation of PPAR*δ* by ginseng increases intracellular calcium, which then results in cardiac troponin phosphorylation. Subsequently, the cardiac contractility is enhanced. Taken together, ginseng enhanced cardiac contractility through an increase in PPAR*δ* expression at the dose that did not modify the heart beating. Thus, ginseng could be developed as a good cardiac agent without the side effect on heart rate.
